# Main 3D Manufacturing Techniques for Customized Bone Substitutes. A Systematic Review

**DOI:** 10.3390/ma14102524

**Published:** 2021-05-12

**Authors:** Javier Montero, Alicia Becerro, Beatriz Pardal-Peláez, Norberto Quispe-López, Juan-Francisco Blanco, Cristina Gómez-Polo

**Affiliations:** Department of Surgery, Faculty of Medicine, University of Salamanca, 37007 Salamanca, Spain; aliciabecerro@usal.es (A.B.); bpardal@usal.es (B.P.-P.); norbert_1404@hotmail.com (N.Q.-L.); jfblanco@usal.es (J.-F.B.); crisgodent@hotmail.com (C.G.-P.)

**Keywords:** 3D scaffold, bone regeneration, tissue engineering, 3D printing

## Abstract

Clinicians should be aware of the main methods and materials to face the challenge of bone shortage by manufacturing customized grafts, in order to repair defects. This study aims to carry out a bibliographic review of the existing methods to manufacture customized bone scaffolds through 3D technology and to identify their current situation based on the published papers. A literature search was carried out using “3D scaffold”, “bone regeneration”, “robocasting” and “3D printing” as descriptors. This search strategy was performed on PubMed (MEDLINE), Scopus and Cochrane Library, but also by hand search in relevant journals and throughout the selected papers. All the papers focusing on techniques for manufacturing customized bone scaffolds were reviewed. The 62 articles identified described 14 techniques (4 subtraction + 10 addition techniques). Scaffold fabrication techniques can be also be classified according to the time at which they are developed, into Conventional techniques and Solid Freeform Fabrication techniques. The conventional techniques are unable to control the architecture of the pore and the pore interconnection. However, current Solid Freeform Fabrication techniques allow individualizing and generating complex geometries of porosity. To conclude, currently SLA (Stereolithography), Robocasting and FDM (Fused deposition modeling) are promising options in customized bone regeneration.

## 1. Introduction

The bone performs many key functions in the body in general and in the mouth in particular, and enables, among other things, the fixation of dental elements. The bone can regenerate spontaneously in healthy conditions, as long as there are walls that limit the defect (self-contained defect); however, the “restitutio ad integrum” is exceptional, and is always performed for small defects. In the mouth, the dimensional loss of the alveolar bone after a tooth extraction or a maxillary intervention is inevitable [1] even if filling biomaterials are used concomitantly [2]. Therefore, the loss of the maxillary bones over time and especially as a result of therapeutic interventions aimed at eliminating dental elements is unavoidable today. Bone shortage is therefore the main challenge that implant surgeons face on a daily basis, and finding a way or system to deal with mandibular atrophy is one of their major concerns [3]. 

Craniofacial bone reconstruction is extremely complex, given the anatomical singularity of each defect, the presence of adjacent neurovascular structures, the risk of infection, etc. Autologous bone grafts are considered the gold standard, as they provide osteoconduction, osteoinduction and certain osteogenesis, and are usually extracted from extraoral (iliac bone, tibia, cranial calotte, etc.) or intraoral (chin, tuberosity, mandibular branch, etc.) areas, depending on the amount required and the surgeon’s preferences [4]. However, they offer certain complications such as poor availability, additional surgeries, morbidity in the donor area, high reabsorption, etc. [5,6]. With respect to the osteogenic capacity of the autograft, we must underline the points classically highlighted by Burchardt [7]. First of all, the number of transplanted cells that survive is usually no more than 10%, a percentage that decreases over time, since cells are extremely sensitive to desiccation: an exposure of more than 30 min would cause total cell death [7]. In addition, the bone trabeculae contain primarily osteocytes, not osteoblasts. This means that the osteogenic potential of the autograft comes mainly from undifferentiated mesenchymal cells of the bone marrow that are transplanted simultaneously or by means of plasma rich in growth factors [7]. Therefore, the cellular response is more dependent on viability and on the cells present in the recipient area stimulated by hormonal factors in the autograft, i.e., it depends more on osteoinduction than on the graft itself [8]. These drawbacks have led to the popularization of the use of biomaterials. In the maxillofacial area, bone defects can be local (or alveolar), regional (affecting a larger sector of the same jaw), or total (affecting the whole jaw), with the extension of the defect being proportional to the complexity of the solution. Alveolar preservation after tooth extraction to facilitate maintenance of bone volume was one of the first applications of biomaterials. This approach is the simplest and can be done with the particulate format with which most of the biomaterials available on the market come. However, partial or total bone defects are still a clinical challenge today. Since the shape of anatomical defects is difficult to replicate intraoperatively at the autologous bone donor site, tissue engineering and three-dimensional biomaterial manufacturing techniques have grown exponentially over the last decade [9] in an effort to address this challenge.

To repair jaw defects, the combination of CAD-CAM systems (Computer Aided Design-Computer Aided Manufacturing) with tissue engineering offers the possibility of manufacturing models, milling/cutting templates and grafts from different materials to guide and scaffold the new regenerated tissue [10]. The ideal material would be one that is biocompatible, osteoconductive, osteoinductive, bioresorbable (allowing it to be biodegraded by the tissue itself in the repair phase), and that has a structure and mechanical properties similar to those of the recipient bone; it should also be easy to use and low-cost [10]. Inspired by the above properties, the following prototype of bone bioblocks is proposed.

- The requirement for it to be resorbable is based on the fact that the bone must be in permanent structural replacement/remodeling, so a nonresorbable element would hinder this process.
- The requirement for structural analogy is also essential, since it has been shown that for biomaterials to be clinically successful, they must have an interconnected macroporous structure (>100 microns in diameter) to promote cell infiltration, bone growth and neovascularization [10]. In addition to macroporosity, it is also necessary for the macroporous structure to have microporosity for optimal cell adhesion, interstitial fluid flow, angiogenesis, etc. [11,12]. The 3D manufacturing technology of biomaterials offers the possibility of replicating the macro and microscopic structure of each bone defect; these biomaterials can be manufactured with porous materials with precise adaptation and internal morphology mimetics [13], which allow them to remain intimately integrated with the native bone [14].

Nowadays we are witnessing an emergence of a multitude of 3D fabrication techniques with multiple materials; some of these techniques are in permanent evolution, others are in extinction, and sometimes they are converging towards technical manufacturing strategies that try to optimize precision, costs and time. Thus, it would be desirable to make a synthesis of the main surviving techniques that could maintain their applicability for customized bone regeneration with resorbable biomaterials. This study aims to review the different methods that exist for manufacturing customized bone regeneration scaffolds with 3D technology.

## 2. Materials and Methods

**Figure 1:**
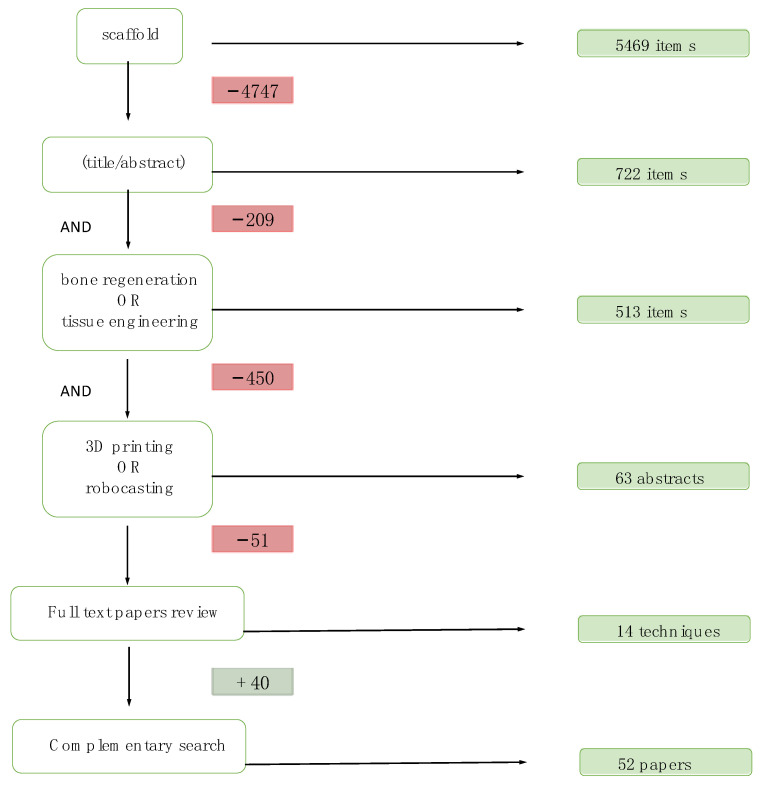
Flow chart of the search strategy.

In May 2020, a systematic review was carried out in PubMed, Embase, Scopus and Cochrane Library with the following Boolean descriptors and operators: scaffold (Title/Abstract) AND (bone regeneration OR tissue engineering) AND (3D printing OR robocasting). After removing duplicates, this resulted in 63 articles that were screened by reading the abstracts, and afterwards 12 articles were finally selected taking into account the following eligibility criteria: articles published in English, focusing on scaffolds fabricated by distinct manufacturing techniques with clinical approach; The exclusion criteria: articles about a single case, regeneration of tissues other than bone or cartilage, papers dealing with bioprinting. By means of a complementary search from those 12 publications, another 40 papers were obtained, making a total of 52 papers (Figure 1).

From the first search, the 14 most relevant techniques were selected, and with the intention of fulfilling the same objective, a search was carried out combining the term bone scaffold for each of the 14 techniques with the following descriptors: (1) Thermally induced phase separation; (2) Solvent Casting; (3) Polymer-Sponge; (4) Sol–Gel Technique; (5) Gas foaming OR supercritical fluid processing; (6) SLA OR stereolithography; (7) SLS OR selective laser sintering; (8) 3DP OR 3D printing; (9) Multi Jet Fusion OR MJF; (10) FFF OR FDM OR fused filament fabrication OR fused deposition modeling; (11) Multi Head Deposition System (12) DIW OR direct ink OR direct ink writing; (13) Low-temperature deposition manufacturing; (14) Pressure-assisted microsyringe.

## 3. Results

With the aim of carrying out a classification of scaffold fabrication techniques and the materials that can be used for this purpose, a total 52 papers were reviewed, being 15 reviews, 17 in vitro studies and 20 in vivo studies (i.e., 16 on animals—with the New Zealand rabbit as the predominant animal—and 4 on human patients). Some of these works focused on the study of biomaterials (Table 1) while others were devoted to an in-depth analysis of fabrication techniques (Table 2), their regenerative efficacy and/or their mechanical properties.

By analyzing the information in Table 1 and Table 2, it can be said that the most widely used materials have been bioceramics, mainly calcium phosphate [23,25,27,29,30,33,34,37,38,46,48,49], although Bioglass has also been the subject of numerous studies [24,26,28,42,43,47]. This is mainly due to their chemical composition, which is similar to the natural bone, showing high biocompatibility and degradability. These bioceramics have been reported to be used independently [23,24,25,26,29,33,34,38,43,49], combined with other bioceramics [46] and doped with materials such as Mg [27,28,30], Sr [30] or Zn [42], bone morphogenic protein BMP-2 [47,48] or collagen [37] (Table 1 and Table 2). The viability of this type of materials is supported by studies carried out on both animals (Beagle dogs [29], rabbits [27,28,48,49] and rats [29,30,37,47]) and humans [33,34], which guarantees their effectiveness over materials that have only been studied on animals. 

Polymers have also been used as a manufacturing material for 3D structures for bone regeneration. They were used individually, mainly in the early years of development of additive manufacturing technologies (1984) [15,17,52], and more recently associated with nanoparticles [45], antibiotics [39], and other substances such as peptides and dopamine [16]. Their efficacy and biocompatibility have been proven in in vivo studies with experimental animals such as Beagle dogs [17] and New Zealand rabbits [16].

Finally, the use of bioceramics combined with polymers (biocomposites)—since the properties of both types of materials are added together—has been shown to provide greater advantages for bone regeneration, from impression accuracy [18] to compressive strength [19,22] and new bone formation [19,20,21,22,31,32,44]. To date, the biocompatibility of these composite materials has been demonstrated in in vitro [18,19,20,44] and in vivo studies with experimental animals only [21,22,31,32,40]. Biocomposites can result from the polymer-ceramic bonding of calcium phosphate (HA or TCP) or bioactive polymer-glass, facilitating cell adhesion and proliferation [20,32] and, as a result, bone regeneration/reparation [15]. In this regard, it has recently been reported that the incorporation of hydroxyapatite (HAP) into a biodegradable polymer (i.e., poly l-lactic acid) (PLLA) matrix exhibit bioactivity and osteoconductivity showing excellent bone defect repair capacity with the formation of abundant new bone tissue and blood vessel tissue [53]. Moreover, authors such as Gendviliene I [18] proved that biocomposites achieved higher printing accuracy than pure polymers. However, biocomposites reduce the mechanical resistance of pure polymers to some extent [21]. In contrast to the previous case, Lin YH [19] showed that adding CSi to the PCL scaffold increased mechanical resistance as well as osteo-regenerative capacity.

With regard to the scaffold fabrication techniques, as we can see in Table 1 and Table 2, except for two studies that used solvent casting [31] and thermally induced phase separation (TICS) [32] and three others that applied the combined lost-wax technique [17,25,29], the rest of studies used additive manufacturing techniques. The techniques with the highest number of publications are firstly DIW, and specifically robocasting [19,23,26,27,42,43,44,46,47,48,49], followed by FFF [16,18,20,21,28,39,40,41] and 3D printing [22,24,30,36,37,38].

Some authors have compared the outcomes gathered by several techniques. Silva [50] showed that the precision of 3D printing and SLS was comparable and acceptable in both cases; Salmi [51] highlighted the PolyJet over the previous two in terms of precision, and Tagliaferri [52] indicated that, among the FFF, SLS and MJF, the FFF was the less convenient option due to the high printing time and environmental impact, which was minimal with MJF (Table 2). Within the additive manufacturing methods, in this review of the literature we found that some of them, such as stereolithography [33,34] and selective laser sintering [35], have been shown to be successful with regard to bone regeneration through studies in humans, which are the most relevant in terms of practical clinical effects. On the other hand, the success of today’s most promising technologies, such as direct ink writing or, more specifically, robocasting, 3D printing or filament extrusion, is based solely on the results from experimental animals.

Seven of the review papers studied in this article [54,55,56,57,58,59,60] focused on the analysis of the properties, advantages and disadvantages of the different materials that have been and are currently used for bone regeneration. On the other hand, five of them [61,62,63,64,65] were dedicated to classifying and studying the different methods for manufacturing customized structures for bone regeneration and how these techniques have evolved over the years. The effect of the pore size on both the biocompatibility and the mechanical strength of ceramic scaffolds needs to be checked in vivo [66,67].

Regarding the ability to customize the scaffold porosity and pore size, Table 3 summarizes the average porosity and macropore sizes obtained with distinct techniques and materials reported in the studies described in Table 1 and Table 2.

With the FFF technique (fused filament fabrication) the macropore size usually ranges between 200 and 350 μm [16,18,20,21,28,40]. Nevertheless, a great variability of pore size was observed with DIW (from 75 μm [23] to 1200 μm [42]) and 3D printing (from 10 μm [36] to 1000 μm [30]). With respect to the average porosity, this parameter is not always reported in the reviewed literature [17,19,20,24,35,37,39,40,41,44,45,48,50,51,52] although it used to range around 40–50% [16,18,23,27,28,30,37,38,47,49].

## 4. Discussion

**Figure 2:**
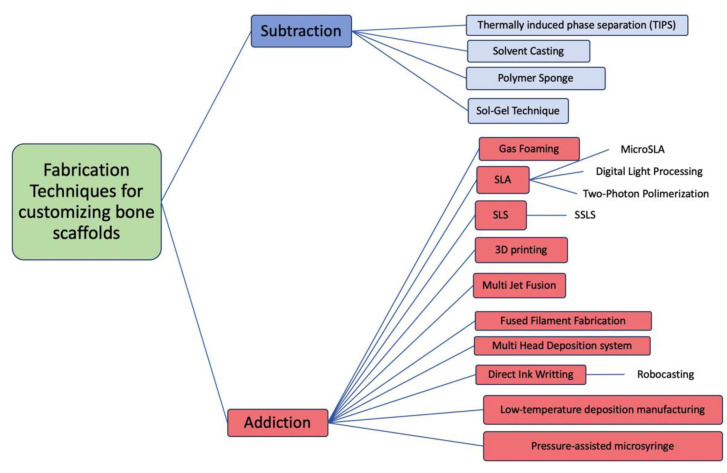
Diagram classifying the main scaffold fabrication techniques according to the manufacturing method.

The techniques used for the fabrication of bone scaffolding can be divided, according to the fabrication method, into Subtraction and Addition (Figure 2). Furthermore, depending on the degree of manual versus computer control in the design and manufacturing process, the techniques may also be classified as conventional (less computerized) or current techniques (more computerized). The conventional techniques had the common problem that the pore architecture cannot be customized, so it is very difficult to control the size of the pores as well as achieving their controlled interconnection. According to Thavornyutikarn [61], these conventional techniques are mostly incapable of producing fully continuous interconnectivity and uniform pore morphology within a scaffold. Most of the conventional techniques manufacture by subtraction. By contrast, the current additive manufacturing techniques, also called Solid Freeform Fabrication Techniques (SFF), offer the possibility of individualizing scaffolds and generating complex geometries with controlled porosity.

### 4.1. Subtraction Techniques

These include all the techniques in which the porous scaffold is obtained after the removal of part of the material from an initial solid or liquid uniform block. Within this group, only conventional techniques are found.

#### 4.1.1. Solvent Casting

A mixture of polymer and ceramic particles is dissolved in an organic solvent and this solution is melted and put into a mold. Afterwards, the solvent is evaporated, leaving a porous scaffold [61]. A variant of this technique is solvent casting + particulate leaching [31], in which the solution mentioned above is used but, in addition, porogen particles are added. After the evaporation of the organic solvent, the scaffold is placed in water or another solvent capable of removing these particles, which generates a higher porosity, with interconnected pores and rough surfaces [31]. The main advantage of this method is that the preparation process is easy and does not require expensive equipment. However, this technique can only form scaffolds of simple shapes (flat sheets and tubes), and the residual solvents left in the scaffold material could be harmful to cells and tissues [61].

#### 4.1.2. Thermally Induced Phase Separation (TIPS)

An organic solvent is used to create the polymer dissolution. In this case, the solution, once introduced into the mold, cools down causing the solvent to solidify and leave spaces among the polymers. The solvent is then evaporated by sublimation, and a porous scaffold is obtained [61]. By means of this technique, a great variety of scaffolds with high porosity can be generated by modifying variables such as the type of polymer and solvent, the polymer concentration, and the phase separation temperature [68,69].

The disadvantages of the two techniques mentioned above (solvent casting and TIPS) [61] are that only simple-shaped scaffolds can be made and that the residual organic solvent could denature proteins and therefore be harmful to biological cells and tissues. In addition, only polymeric structures can be manufactured and are therefore affected by the characteristic shrinkage of these materials [61].

#### 4.1.3. Polymer-Sponge

Starting from a ceramic solution in a suitable solvent (water or alcohol), charges of sucrose, gelatin or PMMA (polymethylmethacrylate) are added so that, as these compounds evaporate during sintering, they will create porosities forming so-called green bodies [70]. Furthermore, it has been described that the addition of polysaccharides increases the resistance of the scaffold [70]. The formation of green bodies can be classified according to the process, since different geometries and porosities are obtained with each one. The main advantage of this also named replication technique relies on the ability to form uniform dispersion of ceramic powder within a template, resulting in controllable pore size, high porosity and well-interconnected scaffolds. However, the equipment needed is quite expensive and the process is time consuming [61].

#### 4.1.4. Sol–Gel Technique

Sol–gel is a chemical route that begins with the synthesis of a colloidal suspension of solid ceramic particles that is called sol. The sol is subjected to a hydrolysis and condensation process that results in the formation of a solid within the solvent, which is called gel [71]. The solvent is extracted from the gel by simply allowing it to rest at room temperature for a period of time, called ageing, during which the gel will shrink by expelling the residual solvent, resulting in a highly porous scaffold [71]. Regarding the main advantages and disadvantages, it should be mentioned that the biodegradability of the structures is satisfactory, and a great variability of forms can be obtained; however, they have low mechanical resistance [70,72,73,74,75,76].

### 4.2. Addition Techniques

These include all those techniques in which the porous geometry of the scaffold is achieved by adding matter, usually layer by layer, without using organic solvent. This group includes both conventional and new techniques.

#### 4.2.1. Gas Foaming/Supercritical Fluid Processing

Mooney developed this conventional technique in 1996 [77] with the aim of eliminating the need for organic solvents and their drawbacks. The polymer is introduced into a chamber and saturated with high pressure CO_2_. The pressure is then rapidly lowered, causing a situation of gas-polymer thermodynamic instability that ends with the formation of pores [77]. Parameters such as temperature, pressure, degree of saturation and speed of depressurization influence the morphology and size of the pores. This technique has the disadvantages of forming closed, noninterconnected pores and a smooth, nonporous surface layer of the scaffold [61]. In addition, it requires excessive heat for its realization [61].

#### 4.2.2. SLA/Stereolithography

This was the first additive manufacturing technique to be introduced in dentistry. It was developed and patented by Chuck Hull in 1984 with the Stratasys company (Eden Prairie, MN, USA). This technology consists of a tank of photosensitive liquid resin, a moving platform and an ultraviolet laser which, when impacted on the resin, will create a solid layer of it. The scaffold is created layer by layer as follows: once the first layer has been made, the platform will descend leaving a new surface of liquid resin that will be polymerized by light creating a second layer, and so on until the scaffolding is complete. At that point, the uncured resin is removed, and the scaffold is subjected to UV light to complete the cure [78,79,80].

Elomaa et al. [81] used degradable polymers as a material and obtained structures with 70–90% interconnected pores. SLA technology can also be used with bioceramics and glass. It was Chu who first described its use with ceramics [82,83,84]. The suspension of ceramics and/or glass in resin has a high density, which makes the SLA process difficult, so some researchers [85,86,87] developed a process combining the SLA technique and casting. The composite fabrication process using SLA is difficult due to the high viscosity of the polymer/ceramic suspensions [88], so this technology has not been widely used with this material.

The SLA technology was the first to create reproducible scaffolds with high dimensional accuracy (up to 50 microns) and surface quality [88,89]; however, it has many drawbacks. It requires expensive machinery, support structure during manufacture, and scaffolding manufacturing time is slow, depending on the size and resolution required. An inherent problem in the process is also shrinkage during sintering. Added to this is the logistical hurdle: there are a small number of photosensitive resins on the market and many of them are toxic at a cellular level [61], although this is a point that can be overcome over time, for example, by using resins based on vinyl esters that have better biocompatibility [90].

Current SLA technologies based on the original conception concept of SLA, there are different techniques, based on SLA technologies that differ in the method of curing the resin. First, the Micro SLA uses a single photon beam for greater precision. Lee et al. [91] used this technique to make poly propylene fumarate scaffolds and Seol et al. [92] for HA and TCP scaffolds. Both studies, performed in vitro, obtained scaffolds with mechanical properties similar to those of human cancellous bone.

First, the so-called Two-Photon Polymerization uses an ultra-short pulse laser and makes it possible to manufacture scaffolds with nanometric resolution [88,89,90]. Second, the Digital Light Processing uses visible light and creates an entire layer at once. It offers a solution to several of the problems of SLA technology. Its main advantage is the speed of synthesis, in addition to the high lateral resolution (40 microns), the large proportion of solid particles it allows (40–60%), and the absence of expensive equipment such as lasers or a heating chamber [92]. Moreover, it allows the manufacture of ceramic and bioglass scaffolds.

#### 4.2.3. Selective Laser Sintering (SLS)

This technology was developed in 1986 and first marketed in 1992. It consists of a CO_2_ laser that acts on a bed of powder to sinter certain regions of the powder to form a solid first layer. The platform lowers the corresponding layer thickness, and a roller deposits a new layer of powder [78,79,80]. Eshraghi and Das [15] manufactured orthogonal pore PCL scaffolds designed for placement in loaded locations. These scaffolds were precise with respect to the digital design and showed acceptable compressive strength. Other authors, such as Pereira et al. [93], have also found great reliability between the virtual model and the manufactured structure. Manufacturing bioceramics using the SLS technique directly has proved difficult, mainly due to the high heating and cooling speeds associated with the high energy laser used [94,95,96]. However, it is currently in use; for example, Feng P. [97] used a bioceramic powder loaded with titanium nanoparticles to improve the mechanical properties of the scaffold, obtaining a compressive strength of 23 MPa with 58% porosity [97]. The SLS technique has also been used to manufacture composite scaffolds but finding the right process parameters is a challenge: powder composition, laser power, particle size, and temperature [61]. This technique has the great advantage of being the only one capable of manufacturing metal structures (such as titanium and cobalt chrome). For example, F. Mangano [35] made dental implants with a sharp edge to rehabilitate highly atrophic maxillae.

The main advantage of this method is that it makes it possible to create reproducible scaffolds, provides greater dimensional accuracy than the SLA technique (<50 microns) and does not require a support structure. However, as with all techniques, it has its drawbacks. Shrinkage during melting or sintering remains a problem as with SLA technology. In addition, the use of high temperature, which could cause the degradation of biodegradable dust, and the difficulty or impossibility of removing the dust once the scaffold has been manufactured, which could hinder cell proliferation and cause an inflammatory reaction, are the major drawbacks. It should also be noted that the resolution will be limited by the size, shape, and arrangement of the dust particles.

To solve the excess of temperature of SLS and to allow the manufacturing of scaffolds with bioactive and biodegradable materials, Popov et al. developed selective laser sintering by surface (SLSS) [98]. This is a variation of the SLS in which the polymer particles are coated with CO_2_, so the melting is limited to the surface layer, maintaining the nature of the particles inside the polymer during the scaffold manufacture [79,99,100].

#### 4.2.4. 3D Printing (3DP)

This was developed in 1989. In this variant of SLS technology, instead of using a laser, a liquid binder is used on the bed of powder to solidify what would be the first layer of the scaffold. Similarly, once the scaffold has been built, any remaining dust must be removed [78,79,80]. This is the only SFF technique that can use hydrogels for the manufacture of scaffolds. The problem with hydrogels is the poor mechanical properties, which force the structure to be processed later to incorporate monomers or polymers so as to increase the mechanical resistance [37,38,101].

Some authors [38,102] have verified the validity of this technique in vivo for the manufacture of scaffolding, especially with calcium phosphate ceramics. However, the finished pieces require subsequent thermal treatment to improve the mechanical properties [30,36,61]. The manufacture of scaffolds from composite materials is also possible, e.g., Sherwood et al. [103] manufactured PLGA/TCP structures that showed a compressive and tensile strength similar to that of cancellous bone.

As it is a technique that does not require high temperature and works with hydrogels, it allows the incorporation of biologically active molecules or even cells. It allows the manufacture of high consistency scaffolds, without support structures and at high speed, which makes mass production feasible. Despite the high consistency, the bonds formed between particles are weak, so scaffolds have poor mechanical properties, as Jason found in a study showing that calcium phosphate scaffolds made with this technology had significantly lower torsional resistance than allografts [37]. Furthermore, it requires a large particle size, which reduces precision and resolution [61] and, as with the SLS techniques, it has the disadvantage of difficult or impossible removal of uncured dust.

When comparing the printing accuracy of SLS and 3DP technology, Silva et al. [50] found that it was acceptable in both cases, with dimensional errors of 2.1% and 2.67%, respectively, slightly higher in the 3DP technique.

A similar technique called PolyJet consists of the extrusion of liquid resin through multiple nozzles, which as soon as it is deposited on the platform, is cured by ultraviolet light. This technology stands out for its high manufacturing speed and printing precision. In the study by Salmi M [51], where the PolyJet is compared in terms of accuracy to 3D printing and SLS, the PolyJet technique showed significantly more accurate results.

#### 4.2.5. Multi Jet Fusion (MJF)

This is a very new and promising technology developed by HP (Hewlett-Packard) [52]. It is based on numerous nozzles capable of releasing different liquid agents onto the printing surface [52]. On the one hand, they release a liquid binder and, on the other hand, a detailing agent to improve resolution. A lamp then runs over the surface, polymerizing and distributing the heat. Finally, the excess dust is removed by blasting [52]. The use of this technique in the field of dentistry has yet to be developed, but the results found in other materials are promising. One of the major advantages of this technology is that it allows mass production due to its speed of processing: it is capable of manufacturing as many parts as fit into the powder hopper at once. The powder used is very fine, so high density, resolution and precision structures are achieved. The uncured powder is reused for the next print, so the waste of material is minimal. This technique also offers the possibility of using different materials.

#### 4.2.6. Fused Filament Fabrication (FFF)

This has also been referred to as Fused Deposition Modeling (FDM), and it was developed in 1992. This technology synthesizes scaffolds by casting material. The system consists of a substrate platform on which there is a mobile nozzle with a small hole. A filament with the corresponding material is introduced into this nozzle, where it melts and is deposited on the platform, giving rise to a first layer. The platform descends, leaving space for the second layer [77,78]. The first scaffolds created by FDM were made of PCL and showed great biocompatibility with human fibroblasts [104]. A filament composed of a thermoplastic polymer, ceramic powder and a binding agent is used to create bioceramic structures through FDM. The polymer and binder are removed during further processing [105,106]. Finished ceramic parts are sintered to improve their mechanical properties [107,108,109,110]. FDM technology has also been used to manufacture composite scaffolds. The research group of Hutmacher et al. [53,110,111] manufactured scaffolds based on various polymers and calcium phosphates that showed favorable mechanical properties, bioactivity, resorption and increased cell colonization and incorporation of growth factors. The study by Gendviliene I [18] showed that the PLA/10% HA filament printed with a 3D FFF printer produced scaffolds with equal or even better accuracy than those printed with pure PLA filament [18].

This technique has numerous advantages, such as its low cost and the achievement of scaffolds of good structural integrity with minimum material waste. In the *X* and *Y* axes, it has a high precision and versatility in the direction of the materials within each layer (0.5 microns [39]); however, the direction of the *Z* axis is not easily controlled (5 microns) [24,39]. As for the disadvantages, this technique requires high temperature, the scaffold manufacturing process is slow and requires support structures, so it does not allow mass production. For successful printing, the viscosity properties of the materials must be considered when casting.

#### 4.2.7. Multi Head Deposition System (MHDS)

This new FFF-based technology, the MHDS (multi head deposition system) consists of using more than one extrusion head to create a composition from several materials, which can be laid out in the same layer [61]. It requires high temperature; however, Kundu J [112] was successful in manufacturing PCL cartilage regeneration scaffolds + alginate hydrogel with encapsulated chondrocytes by adapting the parameters to maintain cell viability [113]. Another variant of the FDP is the Precision Extruding Deposition. The difference between this technique and conventional FDM is that it employs material in the form of granules which is subsequently melted in a chamber, thus avoiding the need to use filament-shaped materials [114].

#### 4.2.8. Direct Ink Writing (DIW)

This arises from the concept of filament extrusion. Here, instead of starting from a material in the form of a yarn, the starting point is a solution of material, which is extruded through a nozzle, so that scaffolds are manufactured layer by layer. Several different techniques can be identified within this group. The advantages are the same as with the FFF techniques, but some of the disadvantages are overcome: it does not require high temperatures to melt the filament and the properties of the material do not have to be considered when melting it.

Robocasting is probably the most promising of the techniques included in DIW technology. It was developed in 1998 by Cesarano et al. [115]. It allows a highly concentrated suspension to be deposited through a small channel on a nonwetting oil bath. The suspension becomes solid when the water evaporates [116]. This technique has been widely used to manufacture bioceramic structures. For example, Pedro Miranda [23] recommends the use of small dust particles and low-specificity surface area, in addition to using Ca-deficient powders to avoid the transition of TCP from beta to alpha. On the other hand, J. Franco [46] describes the preparation of ceramic-based inks (HA, b-TCP and BCP) using Pluronic F-127 as a hydrogel, which is fluid at 0 °C and gel at room temperature. One of its greatest advantages is that scaffolds made by this technique are more resistant than those made by other methods using the same materials. Many authors have supported this statement after finding satisfactory results in their studies [26,42,43,47].

#### 4.2.9. Low-Temperature Deposition Manufacturing (LDM)

This technique combines Direct Ink Writing technology with the Thermally Induced Phase Separation (TIPS), which is one of the conventional techniques deeply explained elsewhere [61]. The LDM was developed by Li (2011) and fabricated PLGA/TCP scaffolds for alveolar bone repair in 2011 showing good biocompatibility in the attachment and proliferation of human bone marrow mesenchymal stem cells.

In this technique, instead of departing from a filament as in the other FFF techniques, it starts from a solution of the material to be used in a low melting point solvent. The deposition of material must be at very low temperatures to allow the material to solidify when deposited on the platform to form layers [117]. The solvent will then be removed by freeze-drying. Almeida et al. [81] developed scaffolds with this technique, which presented porosity greater than 90%, mechanical properties similar to cancellous bone and good biocompatibility and conductivity.

#### 4.2.10. Pressure-Assisted Microsyringe (PAM)

It was developed in 2002 by Vozzi et al. [118]. In this technique, instead of using heat for extrusion, constant pressure is applied, and instead of using a filament, a solution is used. When the solvent evaporates due to pressure, the material solidifies. The higher the viscosity, the higher the resolution [118,119]. This technique has been widely used for the manufacture of drugs [120,121,122,123] and, to a lesser extent, to make polymer scaffolds [118,119]. The same research group [124,125,126] developed the so-called PAM 2, in which they replaced constant pressure with a mechanical piston as the driving force. As it does not require heat, it allows the incorporation of living cells, which is a great advantage, while its major limitation is the need to use low concentration solutions.

### 4.3. Scientific Support of the Techniques

**Figure 3:**
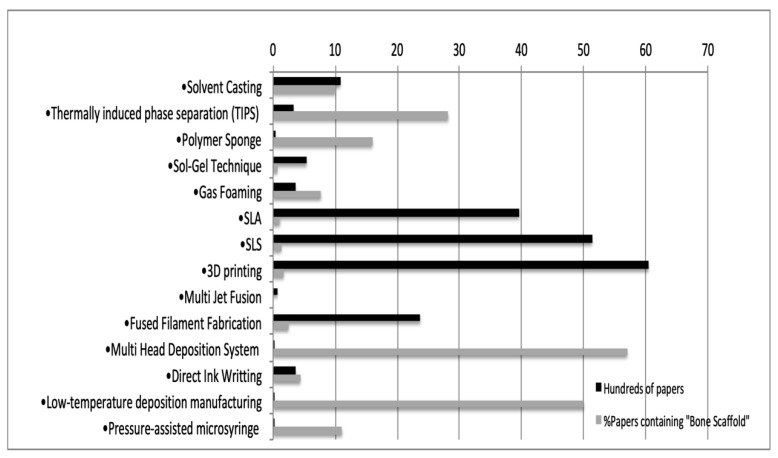
Scientific support of each of the 14 techniques identified as potentially in force regarding the raw number of papers and the percentage of papers containing “bone scaffold”.

The scientific literature supports the 14 techniques to different extents. In Figure 3, it is shown that 3DP, SLS, SLA and FFF are the most studied techniques, being supported by 6043 papers, 5135 papers, 3961 papers and 2368 papers, respectively. However, focusing on the percentage of articles that contained “bone scaffold” within those published papers, the Multi Head Deposition System (57%), the Low-Temperature Deposition Manufacturing (50%) and TIPS (28.2%) stand out as the main techniques applied in bone scaffold manufacturing. However, the Robocasting technique (a variant of DIW) may be considered a promising technique, with 49 out of 70 papers focusing on customized bone scaffolds. Robocasting biocomposites for bone regeneration is increasingly studied in recent years. In this regard, some authors studied the effect of different polymeric coatings (both natural and synthetic), on the mechanical performance of bioceramic robocast scaffolds [126,127], while others focused on the osteostimulative capability of the robocasted biocomposite in animal models [128]. Future experiences will clarify the best choice for customizing bone grafts with the available techniques.

Finally, patents may support most techniques and materials reported in this review; however, we did not search within patent databases and therefore our review is not exhaustive. Consequently, readers should be aware that several current promising techniques or materials could not be retrieved with the search strategy used in the present work, as the patent procedure needs to check the innovativeness/originality of the material/method candidate for patenting.

## 5. Conclusions

There are many techniques for the manufacture of 3D scaffolds. Among them, we can differentiate traditional techniques, which are nowadays practically in disuse in the field of regenerative dentistry, because of the lack of mechanical integrity, as well as the limited capacity to control the internal and external architecture of scaffolds (i.e., pore morphology, pore size, pore interconnectivity and overall porosity). By contrast, the so-called solid freeform fabrication techniques, encompassed under additive manufacturing techniques, overcome the above-mentioned disadvantages. In this regard, SLA, Robocasting and FDM are promising options in customized bone regeneration that enable good mechanical and biological properties throughout the entire scaffold.

## Figures and Tables

**Table 1:** Description of the 16 studies that, both in vivo and in vitro, evaluate the mechanical and regenerative properties of the different materials used to manufacture 3D scaffolds *.

Author/Year	Material	Material and Methods	Fabrication Technique	Results
Eshraghi S. 2010 [15]	Polymer. PCL	3 scaffolds (1D, 2D, and 3D) with different geometries and orthogonal pores, each one more porous than the previous one.	SLS	The structures designed for load bearing locations were accurate with respect to the digital design, and compressive strength was significantly higher in the 1D scaffolds, and the same in the 2D and 3D scaffolds (10.0 ± 0.62 and 0.60 MPa respectively).
Lee SJ. 2019 [16]	Polymer. PCl, PCLD (PCL with polydopamine) and PCLDB (BFP1: bone-forming peptide)	In vitro with human mesenchymal cells and in vivo with New Zealand rabbits.	FFF	Surface treatment with Dopamine and BFP1 considerably increases osteogenesis and angiogenesis.
Xu H. 2010 [17]	Synthetic polymers. PLA/PGA	Eight male Beagle dogs were used.	Lost-wax	The scaffolds were compared with the initial models and proved to be very accurate. The bioblocks demonstrated high biocompatibility when incubated in vitro with mesenchymal bone cells.
Gendviliene I. 2020 [18]	Polymer and composite material. PLA and PLA/HA	Three groups of scaffolds (*n* = 22 each group) were compared, 2 pure PLA with different printers and one PLA/HA.	FFF	Pure PLA frames made with the Pharaoh XD20 printer showed greater accuracy compared to the Ultimaker Original 3D printer, although the highest accuracy was achieved with PLA/HA scaffolds.
Lin YH. 2017 [19]	Composite material. CaSi + PCL	Human mesenchymal cells were used for the in vitro study.	DIW	By adding CaSi to the PCL, compressive strength (5.8 MPa) increased, as did hydrophilia and osteogenic differentiation and angiogenesis.
Roh HS. 2016 [20]	Composite material. PCL + HA + MgO	The scaffolds were treated with oxygen and nitrogen plasma. They were analyzed in vitro with pre-osteoblastic cells.	FFF	The addition of HA and MgO facilitated the initial adhesion, proliferation and differentiation of the cells. The treatment with plasma increased hydrophilicity, enhancing the bioactivity of the scaffolds.
Pae HC. 2018 [21]	Polymer and composite material. PCL + β-TCP.	Ten rabbits with 4 circular calvarial defects of 8 mm each: Control/PCL/PCL + β-TCP/PCL + β-TCP + membrane.	FFF	Compressive strength resistance was higher in PCL (46.7 ± 1.7 N/mm) than in PCL + β-TCP (35.7 ± 3.1 N/mm). PCL/β-TCP + M showed the highest total and new bone volume at 8 weeks and only bioblocks with β-TCP contained new bone (hydrophilicity and conductivity increased)
Kim BS 2018 [22]	Bioceramics. HA and HA + PCL with BMP-2-loaded nanoparticles (NP)	Four rabbits were used with 3 calvarial defects of 6 mm: control/HA/HA + PCL + NP.	3D printing	The PCL-NP coating was useful to incorporate BMP-2/NP to improve bone regeneration, and to improve compressive strength by the PCL (5.10 ± 0.49 MPa).
Miranda P. 2006 [23]	Bioceramics. β-TCP	Structures with different inks, geometries, and nozzle diameters, sintered at different temperatures (1250 °C–1550 °C) depending on the composition of the powder.	DIW. Robocasting	Powders with reduced particle size and a low-specificity surface area were more suitable for manufacturing through robocasting. To avoid TCP transition (from beta to alpha): calcium deficient powders and sintering temperatures below 1125 °C.
Zhou Z. 2014 [24]	Bioceramics. Calcium phosphate mixed with calcium sulphate (CaSO_4_)	The effects of particle size, the CaP/CaSO_4_ ratio and the type of CaP powder (HA/TCP) were measured.	3D printing	Best result with a powder size of 30–110 microns and a higher proportion of CaP with respect to CaSO_4_ (25/75). HA performed better than b-TCP: good print accuracy and compressive strength for no-load defects (1.98 MPa).
Guda T. 2012 [25]	Bioceramics. HA with different porosities and ratio between cortical and trabecular layer.	Six cylindrical samples of each type of 8 mm in diameter and 16 in length	Lost-wax	Although the elastic module did resemble that of human bone, the compressive strength was much lower than that of the trabecular bone. It was also shown that the macropore size of the core does not influence the mechanical aspect.
Eqtesadi S. 2014 [26]	Bioceramics. Bioactive glass 45S5	Compared the mechanical properties of bioglass 45S5 obtained with robocasting against other techniques.	DIW. Robocasting	Compressive strength = 2–13 MPa Robocasting is the best option for 45S5 glass structures with the necessary mechanical properties for their clinical application.
H. Shao. 2018 [27]	Bioceramics. Typical porous bioceramics were compared with wollastonite with Mg-10% (CSiMg_10_).	Alveolar defects were created in the jaws of 32 rabbits. They were sacrificed at 8 and 16 weeks. A total of 64 samples were obtained.	DIW	In vitro, CSiMg_10_ scaffolds were placed in a liquid buffer and showed a slight dissolution, moderate weight loss (7%) and hardly any reduction in bending strength (31 MPa). In vivo, they revealed a significantly higher osteogenic capacity than the TCP, CSi and Bred scaffolds after 16 weeks.
ShaoH. 2017 [28]	Bioceramics. Pure calcium silicate (CSi) and CSiMg_6_.	Structures of different thicknesses by printing in one or double layer and sintering in 1/2 steps. Twenty-four rabbits were used for the in vivo study.	FFF	CSiMg_6_ and two-step sintering showed the best compression and bending strength figures (104/18 MPa). Single layer structures had greater bone formation in the short term (4 weeks), and double layer in the long term (8–12 weeks). The CSi showed greater regeneration. In the CSiMg_6_, regeneration was also acceptable, with the advantage of high fracture resistance.
Lee. YK. 2008 [29]	Bioceramics. Calcium phosphate glass with a significantly lower Ca/P ratio than typical calcium phosphates.	For the in vivo study, the following were used: - Calvarial defects of 60 rats - 12 intraosseous defects from 1 wall of 6 male Beagle dogs.	Lost-wax using polyurethane ester cross-linked sponges.	In vitro, the degree of dissolution and the calcification and mineralization were improved by Calcium phosphate glass. In vivo in rats and dogs, a significant improvement in bone and cement formations was observed with Calcium phosphate glass.
Tarafder S. 2013 [30]	Bioceramics. Bioblocks from β-TCP pure and doped were compared with Sr-Mg.	Twenty-four male rats in which 20 bioblocks of β-TCP pure (Control) and 20 doped with Sr-Mg (study) were placed.	3D printing	The compressive strength of the study was higher than that of the control (12.01 ± 1.56 MPa and 10.95 ± 1.28 MPa respectively). At 12–16 weeks, the bone formed in the control was less mineralized. At 16 weeks, it was mineralized in both bioblocks. Biological performance in vivo was improved by the addition of SrO and MgO.

* Visit the acronyms section at the end of the paper for the description of the material and/or technique mentioned in this table.

**Table 2:** Description of the 22 main research works found after the bibliographic search that, both in vitro and in vivo, evaluate the regenerative efficacy and the mechanical properties of scaffolds obtained with different fabrication techniques *.

Author/Year	Fabrication Technique	Study Design	Materials	Results
Cao H. 2010 [31]	Solvent casting	In vivo: 40 rats with femur defects	Scaffolds made with HA and PGA-betaTCP at different % (1:1 and 1:3) were compared.	The PGA-betaTCP bioblock (1:3) obtained a higher density and new bone formation than the rest 90 days after surgery, as well as a reabsorption rate appropriate to the process.
Yang L. 2019 [32]	TIPS	In vivo: Nine female New Zealand white rabbits were used, and two operations were performed on each.	PLGA and PLGA/bioglass scaffolds were compared.	Both implants had similar porosities (93.926% and 93.048% respectively) while the scaffold with bioglass showed a higher rate of cell adhesion.
Brie J. 2013 [33]	SLA	In vivo: Eight bone implants in 8 patients	Hydroxyapatite	Three types of grafts were designed, two of which were solid and a third had macropores in the areas of attachment to the native bone. After healing, gaps were observed in the massifs on palpation, while the macropores formed a smooth interphase.
Staffa G. 2012 [34]	SLA	In vivo: Sixty patients with large cranial defects.	Hydroxyapatite	None of the patients suffered rejection, spontaneous fracture, or mobilization of the graft and all reported good initial and long-term aesthetics.
Mangano F. 2013 [35]	SLS	In vivo: Five patients with severe mandibular atrophy	Master alloy (Ti_6_Al_4_)	Blade-shaped dental implants were manufactured to rehabilitate atrophic maxillae. After 2 years of follow-up, all the implants were still in function and with good integration and good esthetic results.
Cox SC. 2015 [36]	3D printing	In vitro: They were printed on the *X*-axis and the *Y*-axis for comparison.	HA (50%) and PVOH (polyvinyl alcohol)	Variation in mechanical resistance (0.88 MPA in the *Y* axis and 0.76 MPa in the *X* axis). However, PVOH degradation products were found in the *Y*-axis after the thermal treatment.
Inzana JA. 2014 [37]	3D printing	In vivo: Defects were created in the femurs of 12 female mice: - Allografts (*n* = 4) - 3D scaffold (*n* = 4) - Empty (*n* = 4)	Pure calcium phosphate bioceramic, coated with collagen and embedded in collagen.	The mechanical resistance of all the pure calcium phospate was significantly lower than that of the allografts, although none reached the values of the intact femur (19.4 ± 5.6 N mm). In terms of bone formation, the scaffolds were osteoconductors but poorly osteoinductors; they did not completely cure the defect on their own.
Torres, J. 2011 [38]	3D printing	In vivo: Eight New Zealand rabbits in which a total of 16 bone blocks were placed in calvaria.	Monetite (calcium phosphate ceramic)	The surgical procedure was easy and fast. After 8 weeks, the 4 and 3 mm high blocks were fused to the bone surface and filled with 35% and 41% respectively of newly formed bone.
Lee JH. 2020 [39]	FFF (MHDS)	In vitro: Human osteoblasts were used to determine compatibility and appropriate drug concentration.	PCL with rifampicin	Successful scaffolds were developed for the treatment of osteomyelitis by printing at 60 °C so as not to alter the properties of the drug.
Zheng P. 2019 [40]	FFF	In vivo: Scaffolds were placed in 9 female New Zealand rabbits with femoral defects for osteochondral regeneration	PCL-HA coated with mesenchymal stem cells and chondrocytes	This PCL-HA scaffold promoted increased joint cartilage repair compared to the PCL-HA unseeded control scaffolds, thus concluding that the use of chondrocytes and mesenchymal cells stimulates cartilage regeneration.
Lethaus B. 2012 [41]	FFF	In vivo: Manufacture of mandibles prior to resection in 20 patients to pre-form the reconstruction plates.	Not applicable	They demonstrated great accuracy and significantly facilitated the process.
Roohani-Esfahani SI. 2016 [42]	DIW. (Robocasting)	In vitro: Highly porous hexagonal architectural glass-ceramic structures were manufactured.	Bioglass (Sr doped with Ca_2_ZnSi_2_O_7_(HT))	Thanks to the optimization of the geometry, a compressive strength of 100–110 MPa and a high fatigue and flexural strength (30 MPa) were achieved: 150 times more than polymer and composite bioblocks and 5 times more than other made of bioceramics with similar porosity but different geometry.
Fu Q. 2011 [43]	DIW. (Robocasting)	In vitro: Inks with 30% powder with low viscosity at 0° and high viscosity at 40 °C were used. An SBF was used to evaluate the properties.	Bioglass 6P53B	Compressive strength, with 60% porosity, of 136 ± 22 MPa, which remained above the values of the trabecular bone (77 MPa) after being immersed for 3 weeks in a simulated body fluid.
Hong SJ. 2009 [44]	DIW (Robocasting)	In vitro: Rat bone marrow stromal cells (rBMSC) were used.	PCL and PCL/HA	The HA-PCL scaffold with robotic dispensing has potential applications as a bioactive matrix. Despite showing limited cell adhesion, it proved to stimulate osteogenic differentiation.
Ma C. 2019 [45]	DIW and DIW/Solvent casting	In vitro: 3D and 2D scaffolds (membranes) were manufactured. All three groups of materials were cultured with fibroblasts in vitro.	PLLA, PLLA with tubular and spherical polypyrrole nanoparticles.	The nanoparticles increased the tensile strength (membranes from 100 to 250 MPa). Biocompatibility was satisfactory in all cases. Using these techniques, the 3D and 2D scaffolds were successful in optimizing the physiological microenvironment, which could be adapted to regenerate different tissues.
Franco J. 2010 [46]	DIW. Robocasting	In vitro: The ink was created with 30–50% powder and Pluronic F-127 as hydrogel.	HA, b-TCP and HA/b-TCP with Pluronic F-127 solutions	A high pluronic content adds stability to the ink but, as a result, creates larger microporosities and less mechanical resistance.
Liu X. 2013 [47]	DIW Robocasting	In vivo: 30 male Sprague-Dawley rats in which calvarial defects were created in each parietal bone.	Bioglass 13-93. They were introduced into K_2_HPO_4_ to create a superficial layer of HA, or BMP-2 was added to the bioglass.	Both strategies both individually and in combination proved to be effective in improving bone regeneration of calvarial defects.
Abarrategi A. 2012 [48]	DIW Robocasting	In vivo: - Rabbit muscle. Six rabbits. - Rabbit bone (leg) Cinco rabbits. Sample collection after 3 weeks. - Pig maxilla (palate). Eight pigs. Sample collection after 3 months.	Bioceramics (HA/betaTCP) with BMP-2 protein (study). As a control: scaffolds without BMP-2 in muscle and BioOss in bone.	In muscle: - Controls. Muscle growth (osteoconduction) - Study. Bone growth (osteoinduction). In bone: Similar results between the study scaffolds and the BioOss were obtained; the scaffolds also presented the advantages of being customized and facilitating surgical insertion.
Tovar N. 2018 [49]	DIW. (Robocasting).	In vivo: Fifteen New Zealand rabbits with radial diaphysis defects. They were analyzed at 8 (*n* = 9), 12 (*n* = 3) and 24 (*n* = 3) weeks.	β-TCP	At 12 and 24 weeks, a large amount of bone was found which led to the regeneration of the marrow space. The amount of scaffold was much higher at 8 than at 12 and 24 weeks, between which there was not much difference.
Silva DN. 2008 [50]	SLS and 3D printing	In vitro: Dry human skulls were used to measure and compare the accuracy of the techniques.	Gypsum powder and water were used as a binder.	The SLS and 3DP printing accuracy was acceptable; an error of 2.1% and 2.67% was obtained respectively when comparing the real skulls with those manufactured via these techniques from the CT.
Salmi M. 2013 [51]	SLS, 3DP and PolyJet	In vitro: Dry human skulls were used to measure and compare the accuracy of the 3 techniques with a new measurement method.	Not applicable	Using the method used (based on positioning 6 balls on the 3D model, measuring the distance between them and determining their midpoint), they found considerably greater accuracy (0.18 ± 0.12%) with PolyJet technology as compared to SLS (0.79 ± 0.26%) and 3DP (0.67 ± 0.26).
Tagliaferri V. 2019 [52]	FDM, SLS and MJF were compared.	In vitro: Six objects with different geometries were selected for analysis.	(Polyamide) Nylon 12 (in powder form for SLS and MJF and in filament form for FDM).	SLS and MJF have the advantage that several components can be manufactured at the same time. FDM technology has the greatest limitations due to the high time and cost, as well as the high environmental impact, which was minimal with the MJF technique.

* Visit the acronyms section at the end of the paper for the description of the material and/or technique mentioned in this table.

**Table 3:** Summary of the average porosity and macropore sizes reported in the studies described in Table 1 and Table 2.

Author/Year	Material	Fabrication Technique	Porosity (%)	Macropore Size (μm)
Eshraghi S. 2010 [15]	Polymer. PCL	SLS	37–55	700 μm
Lee SJ. 2019 [16]	Polymer. PCL, PCLD and PCLDB	FFF	50	300 μm
Xu H. 2010 [17]	Synthetic polymers. PLA/PGA	Lost-wax	Not specified	Not specified
Gendviliene I. 2020 [18]	Polymer and composite material. PLA and PLA/HA	FFF	48	350 μm
Lin YH. 2017 [19]	Composite material. CaSi + PCL	DIW	Not specified	500 μm
Roh HS. 2016 [20]	Composite material. PCL + HA + MgO	FFF (PED)	Not specified	300 μm
Pae HC. 2018 [21]	PCL y β-TCP.	FFF (PED)	30	240–260 μm
Kim BS 2018 [22]	Bioceramics. HA and HA + PCL	3D printing	65–67	Not specified
Miranda P. 2006 [23]	Bioceramics. Beta-TCP	DIW. Robocasting	45	75 μm
Zhou Z. 2014 [24]	Bioceramics. (CaSO_4_)	3D printing	Not specified	1–100 μm
Guda T. 2012 [25]	Bioceramics. HA with cortical and trabecular layers.	Lost-wax	60.1–71.7	Outer layers 200–250 μm Inner layers 340–450 μm
Eqtesadi S. 2014 [26]	Bioceramics. Bioactive glass 45S5	DIW. Robocasting	60–80	287 × 820 μm
H. Shao. 2018 [27]	Bioceramics. Typical porous bioceramics were compared with wollastonite with Mg-10% (CSiMg_10_).	DIW	TCP: 57.3 ± 4.4 CSi: 56.6 ± 5.3 CSiMg_10_: 51.2 ± 4.6% Bred: 61.2 ± 5.2%	TCP: 302 μm × 261 μm CSi: 304 μm × 257 μm CSiMg_10_: 313 μm × 259 μm Bred: 318 μm × 255 μm
ShaoH. 2017 [28]	Bioceramics. Pure calcium silicate (CSi) and CSiMg_6_.	FFF	CSi: - SL 58.3 ± 1.9 - DL 59.2 ± 2.3 CSiMg_6_: - SL 53.1 ± 1.4 DL 53.5 ± 1.6	CSi: - SL 305 μm × 132 μm - DL 280 μm × 316 μm CSiMg_6_: - SL 277 μm × 130 μm DL 270 μm × 304 μm
Lee. YK. 2008 [29]	Bioceramics. Calcium phosphate glass	Lost-wax.	80.7–90.3	From 371.6 ± 12.8 μm to 703.2 ± 17.1 μm
Tarafder S. 2013 [30]	Bioceramics. Bioblocks from β-TCP pure.	3D printing	49.44 ± 4.64	350 μm
Cao H. 2010 [31]	Biocomposites HA and PGA-β-TCP	Solvent casting	88.4–93.6	483.3–504.2 μm
Yang L. 2019 [32]	PLGA and PLGA/bioglass.	TIPS	93–94	1–7 μm
Brie J. 2013 [33]	Hydroxyapatite	SLA	50–70	300–550 μm
Staffa G. 2012 [34]	Hydroxyapatite	SLA	70	150 μm
Mangano F. 2013 [35]	Master alloy (Ti_6_Al_4_)	SLS	Not specified	Not specified
Cox SC. 2015 [36]	HA (50%) and PVOH (polyvinyl alcohol)	3D printing	55	10–60 μm
Inzana JA. 2014 [37]	Pure calcium phosphate bioceramic, coated with collagen.	3D printing	Not specified	50–70 μm
Torres, J. 2011 [38]	Monetite (calcium phosphate ceramic)	3D printing	44	Not specified
Lee JH. 2020 [39]	PCL with rifampicin	FFF(MHDS)	Not specified	Not specified
Zheng P. 2019 [40]	PCL-HA	FFF	Not specified	200 μm
Lethaus B. 2012 [41]	Not applicable	FFF	Not specified	Not specified
Roohani-Esfahani SI. 2016 [42]	Bioglass (Sr doped with Ca_2_ZnSi_2_O_7_(HT))	DIW. (Robocasting)	50, 55, 60 and 70	450, 550, 900 and 1200 μm
Fu Q. 2011 [43]	Bioglass 6P53B	DIW. (Robocasting)	60	200 μm
Hong SJ. 2009 [44]	PCL and PCL/HA	DIW (Robocasting)	Not specified	500 μm × 500 μm
Ma C. 2019 [45]	PLLA, PLLA with tubular and spherical polypyrrole nanoparticles.	DIW/Solvent casting	Not specified	100 μm
Franco J. 2010 [46]	HA, b-TCP and HA/b-TCP with Pluronic F-127 solutions	DIW. Robocasting	10–40	200 μm × 180 μm
Liu X. 2013 [47]	Bioglass 13-93 with layers of HA.	DIW Robocasting	50	300 μm
Abarrategi A. 2012 [48]	Bioceramics (HA/betaTCP)	DIW Robocasting	Not specified	225 μm × 400 μm
Tovar N. 2018 [49]	β-TCP	DIW. Robocasting.	58.6 ± 3.0	400 μm
Silva DN. 2008 [50]	Gypsum powder.	SLS and 3D printing	Not specified	Not specified
Salmi M. 2013 [51]	Not applicable	SLS, 3DP and PolyJet	Not specified	Not specified
Tagliaferri V. 2019 [52]	Polyamide.	FDM, SLS and MJF.	Not specified	Not specified

## Data Availability

No new data were created or analyzed in this study. Data sharing is not applicable to this article.

## Acronyms

3DP	3D printing
BMP-2	bone morphogenic protein-2
CAD-CAM	Computer Aided Design-Computer Aided Manufacturing
DIW	Direct ink writing
FDM	Fused deposition modeling
FFF	Fused filament fabrication
GF	Gas foaming
HA	Hydroxyapatite
LDM	Low-temperature deposition manufacturing
MJF	Multi Jet Fusion
PAM	Pressure-assisted microsyringe
PCL	Polycaprolactone
PEEK	Polyetheretherketone
PGA	Polyglycolic acid
PLA	Polylactic acid
PLLA	Poly l-lactide
PLG	Polyglycolic acid
PLGA	Poly(lactic-co-glycolic acid)
PVOH	Poly(vinyl)alcohol
S-G	Sol–gel technique
SBF	Simulated body fluid
SFF	Solid-Free-Fabrication
SC	Solvent casting
SLA	Stereolithography
SLS	Selective laser sintering
SSLS	Selective laser sintering by surface
TCP	Tricalcium Phosphate
TIPS	Thermally induced phase separation
